# Assessing photo-specific social media, body dissatisfaction, anxiety, and depression: a nationwide cross-sectional study on adolescents and young adults

**DOI:** 10.1097/MS9.0000000000004535

**Published:** 2026-01-28

**Authors:** Bakhtawar Latif, Zoya Rehman, Faisal Ahsaan, Muddassir Khalid, Maham Faisal, Ghulam Dastgeer, Zainab Bibi

**Affiliations:** a Department of Community Medicine, Sheikh Khalifa bin Zayed Al-Nahyan Medical College, Lahore, Pakistan; b Department of Community Medicine, Nishtar Medical University, Multan, Pakistan; c Department of Psychology, Bahria University Lahore Campus, Lahore, Pakistan

**Keywords:** body dissatisfaction, Instagram, mental health, public health, Snapchat, social media, TikTok, young adults

## Abstract

**Background:**

The rise of photo-specific social media platforms has significantly impacted body image and mental health. Although studies have shown a link between social media use and body dissatisfaction, most prior research has focused on adult and Western populations, leaving a gap in understanding these relationships among adolescents and young adults in non-Western contexts like Pakistan. Furthermore, the specific roles of active photo-editing behaviors and exposure to influencer content remain under-investigated.

**Methods:**

This cross-sectional study investigated the prevalence of body dissatisfaction, anxiety, and depression among social media users in Pakistan aged 16–25 years. A total of 511 participants completed a survey assessing social media engagement including time spent on Instagram, TikTok, and Snapchat, usage of filters and photo-editing tools, and exposure to beauty and fitness content. The Body Shape Questionnaire (BSQ-8C), the Sociocultural Attitudes Toward Appearance Questionnaire (SATAQ-4), GAD-7, and PHQ-9 scales were used. Data analysis was conducted in SPSS and included descriptive statistics, non-parametric independent sample test, and multinomial regression analysis.

**Results:**

Out of 511 participants, 212 (41.5%) were in the 21–23 years age category, and 315 (61.6%) were female. Snapchat was the most frequently used platform for photo-filters (178, 51.8%). The median (IQR) BSQ-8C score was 21 (19). Significant positive correlations were observed between BSQ-8C, SATAQ-4, GAD-7, and PHQ-9 (all *P* < 0.001). Multinomial regression identified gender and the perceived importance of photo-filter use as significant predictors of body dissatisfaction. The negative coefficients indicate that being female (*B* = −3.778, *P* = 0.008) and attributing greater importance to filters (*B* = −6.383, *P* = 0.029) were associated with significantly higher body dissatisfaction.

**Conclusion:**

This study provides evidence from a Pakistani cohort that engagement with photo-specific social media is associated with body dissatisfaction and poorer mental health, with females being particularly vulnerable. The finding that the importance placed on photo filters is a key predictor highlights a critical risk factor. These results emphasize the need for targeted public health strategies, including media literacy programs that critically address photo-editing and curated content and accessible mental health support for young people.

## Introduction

In this modern digital era, social media has become a central part of our lives. With the increasing use of photo-specific social media platforms like Instagram, TikTok, and Snapchat, where users are exposed to idealized and edited images, a question about their impact on the minds and habits of the youth arises. These platforms allow users to curate visual content and share photographs publicly, hence playing a crucial role in shaping people’s perceptions of themselves and their bodies, while setting unrealistic beauty standards.

Body dissatisfaction is a negative self-evaluation of one’s body size, shape, or appearance that has been linked to various adverse outcomes, including low self-esteem, depression, and eating disorders[1]. By comparing oneself to these often unattainable and heavily edited images, feelings of inadequacy and self-consciousness can intensify, especially among adults frequently exposed to these platforms.

Researchers worldwide have highlighted the association of body dissatisfaction with social media use. In a study, M. Scully and L Sword revealed that the time spent being engaged in social comparisons significantly mediated the relationship between online appearance-related activity and body dissatisfaction[2]. Additionally, in a study, Brown and Tiggemann found that young adults who frequently viewed fitness-related content on Instagram reported higher levels of body dissatisfaction and a greater drive for thinness[3]. Cohen *et al* demonstrated a strong association between social media usage and body image concerns, especially among women[4]. Furthermore, the use of filters and photo-editing tools exacerbates these issues by promoting unrealistic body ideals[5].

Prolonged social media use has also been frequently associated with poor mental health and according to a study by Lopes LS, a strong relationship was found between social media use and depression or anxiety which was often related to problematic social media use[6].

With this in mind, there remains a need for more comprehensive research focusing specifically on the impact of photo-specific social media on body dissatisfaction along with its association with anxiety and depression among adolescents and young adults. Most prior studies have mostly targeted adult age groups, thus providing scanty knowledge about the effects of photo specific social media applications on young people. This study investigates the association between the use of platforms such as Instagram, Snapchat, and TikTok and levels of body dissatisfaction, anxiety, and depression among Pakistani adolescents and young adults. It further questions the role of visual filters, photo-editing tools, and being exposed to beauty and fitness influencers in determining such psychological consequences. By focusing on individuals aged 16–25 years, the study aims to provide culturally relevant evidence to guide preventive strategies promoting media literacy, authenticity, and body acceptance in the modern digital landscape.

This cohort/cross-sectional study has been reported in line with the STROCSS guidelines^[^7^]^.

## Methods

This research utilized a cross-sectional study. After seeking IRB (Institutional Review Board) approval, TERC ID: TERC/SC/INT/2024/254, the data were collected by sending out online survey forms to residents of the city of Lahore, Pakistan, over 1.5 months till the required number of responses was attained. The sampling technique employed was convenience sampling.

Individuals under 16 or over 25, and users who engage with the specified platforms for less than 1 hour per day, were excluded. To minimize confounding, we also excluded individuals with a clinically diagnosed history of mental health conditions (e.g., major depressive disorder, anorexia nervosa, and bulimia nervosa) as reported by the participants.

The primary outcomes in this study were body dissatisfaction, measured using the *Body Shape Questionnaire (BSQ-8C)*[8]. Responses were typically scored on a six-point Likert scale ranging from 1 (“Never”) to 6 (“Always”). Higher scores indicated greater body dissatisfaction. The Sociocultural Attitudes Towards Appearance Questionnaire (SATAQ-4)[11] was used to evaluate the influence of societal and social media pressures on body image. Mental health outcomes, including anxiety and depression, were assessed through the *GAD-7*[9] *and PHQ-9*[10] scales. Responses were scored on a four-point scale from 0 (“Not at all”) to 3 (“Nearly every day”). A total score was calculated, with higher scores indicating higher levels of anxiety. A pretest comprising 30 responses was done to validate these questionnaires in our population. The Cronbach’s α values were as follows: BSQ-8C 0.94, STATQ-4 0.903, GAD-7 0.812, and PHQ-9 0.84.

**HIGHLIGHTS**

- Photo-filter use on social media predicts body dissatisfaction.
- Female users are more vulnerable to body image concerns.
- Strong link found between body dissatisfaction, anxiety, and depression.
- Snapchat was the most used platform for photo-filters among youth.
- Study focuses on a young, non-Western demographic (Pakistan).

A self-developed questionnaire was utilized to assess social media use across platforms such as TikTok, Instagram, and Snapchat. It measured the duration of daily usage (1–2 hours, 3–4 hours, > 5 hours, or non-use), the type of content most frequently viewed (fitness, beauty, lifestyle, fashion, or other), and the frequency of filter or photo-editing tool usage (never, rarely, sometimes, often, always). Additionally, it captured the platforms commonly used for photo editing (Instagram, Snapchat, TikTok, or others) and the perceived importance of photo editing (not important, slightly important, moderately important, and extremely important).

Based on a sample size calculation using OpenEpi, 471 participants were needed for a 97% confidence level, with the calculation guided by population estimates (69.9 million social media users) from a 2025 Kepois team survey^[^12,13^]^. A total of 511 responses were collected, providing adequate power to detect significant associations.

Data analysis was conducted using SPSS version 27. Descriptive statistics outlined demographic and social media usage characteristics. The Kolmogorov-Smirnov test was applied to check the normality of the data. The total score of BSQ-8C, STATAQ-4, GAD-7, and PHQ-9 was not normally distributed. The non-parametric tests were applied to find any significant difference of these total scores among categories of gender and social media platforms (Instagram, TikTok, and Snapchat). Spearman’s correlations examined relationships between BSQ-8C, STATAQ-4, GAD-7, and PHQ-9. Multinomial regression analysis controlled for confounding factors and identified predictors of body dissatisfaction and the sociocultural attitude toward appearance. The *P*-value <0.05 was considered statistically significant.

## Results

The distribution of participants by age group shows that the majority 212 (41.5%) fall between 21 and 23 years, with a smaller proportion in other age groups. A majority of participants were female, 315 (61.6%), with males making up 196 (38.4%) of the study population. Among the social media platforms, Snapchat was the most preferred platform for photo-filter use 178 (51.1%). A total of 102 (19.8%) participants reported using filters often. The rest of the demographics are mentioned in Table 1.

**Table 1:** Demographic details among study participants and social media usage patterns among study participants

	Variable	Frequency	Percentage
Age (years)	16–18	97	19.0
	18–20	95	18.6
	21–23	212	41.5
	24–25	107	20.9
Gender	Female	315	61.6
	Male	196	38.4
	**Variable**	**Frequency**	**Percentage**
Preferred social media platforms for filters	Instagram	178	41.0%
	Snapchat	221	51.1
	TikTok	99	22.9
	Others (Facebook, WhatsApp)	161	37.1
Patterns of filter usage	Always	61	9.8
	Often	102	19.9
	Sometimes	143	30.0
	Rarely	117	23.6
	Never	89	16.7
Types of content viewed	Fitness	214	50.1
	Beauty	193	45.2
	Lifestyle	249	58.2
	Fashion	202	47.2
	Other (food, funny, drama)	200	46.7

Instagram emerged as the most widely used platform, with 449 (87.9%) of respondents actively engaging, nearly half of respondents did not use Snapchat 172 (33.7%) or TikTok 249 (48.7%), reflecting varied engagement across platforms. The platform preference daily usage is given in Table 2.

**Table 2:** Overview of platform preferences and daily usage

Platform	Total usage *N* (%)	1–2 hours daily usage *N* (%)	3–4 hours daily usage *N* (%)	>5 hours daily usage *N* (%)	Non-users *N* (%)
Instagram	449 (87.9%)	169 (33.1%)	156 (30.5%)	124 (24.3%)	62 (12.1%)
Snapchat	262 (51.3%)	213 (41.7%)	60 (11.7%)	66 (12.9%)	172 (33.7%)
TikTok	262 (51.3%)	154 (30.1%)	58 (11.4%)	50 (9.8%)	249 (48.7%)

The analysis of the participants (*N* = 511) revealed that the Median (IQR) score for BSQ-8C was 21 (19). Similarly, the Median (IQR) score on the SATAQ-4 was 18 (12). In terms of psychological well-being, the Median (IQR) score for the GAD-7 was 7 (12), while the Median (IQR) for the PHQ-9 was 11 (11).

As shown in Table 3, significant positive correlations were found between BSQ-8C, SATAQ-4, GAD-7, and PHQ-9 (*P* < 0.001). These findings suggest that individuals who experience higher levels of body dissatisfaction tend to have more negative sociocultural attitudes toward their appearance and are also more likely to experience higher levels of anxiety and depression.

**Table 3:** Correlations between body dissatisfaction, sociocultural attitudes, anxiety, and depression

	BSQ 8C score	SATAQ4 score	GAD7 score	PHQ9 score
BSQ 8C score	1.000	0.751^a^	0.479^a^	0.585^a^
SATAQ4 score		1.000	0.505^a^	0.576^a^
GAD7 score			1.000	0.681^a^
PHQ9 score				1.000

a Correlation is significant at the 0.01 level (two-tailed).

Non-parametric Independent Sample Test was conducted to compare the median score of BSQ-8c, STATAQ4, GAD-7, and PHQ-9 among categories of Instagram, TikTok, and Snapchat usage. The results indicated a significant difference (*P* < 0.05) among groups. Details are mentioned in Figures 1–3.

**Figure 1.**
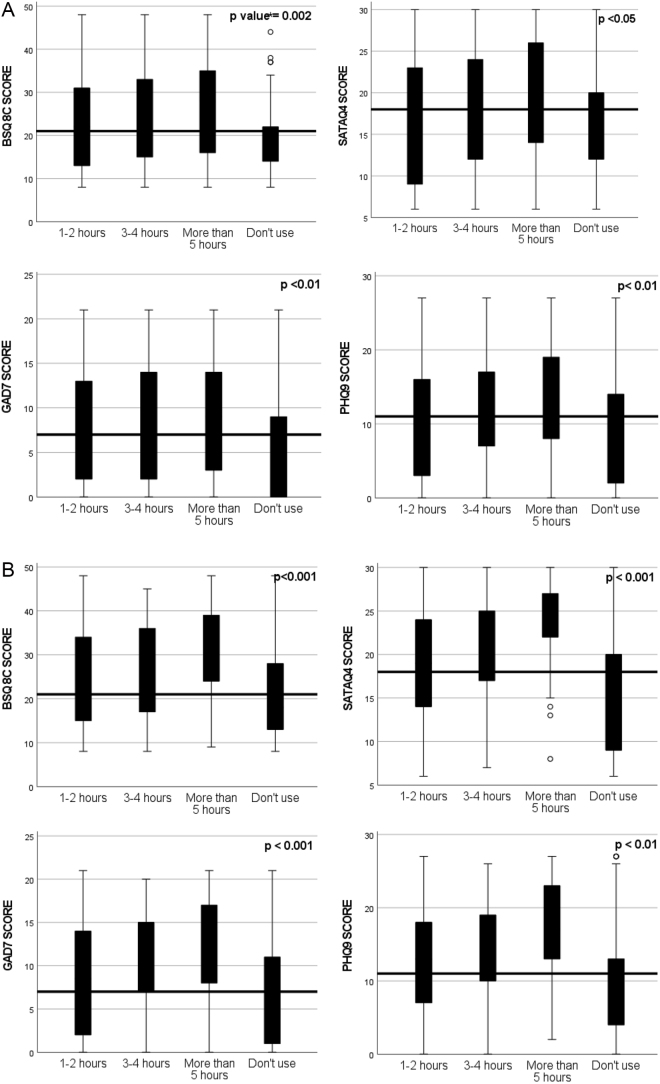
(A) Comparison of total score of BSQ-8C, SATAQ4, GAD-7, and PHQ-9 among categories of instagram usage. (B) Comparison of total score of BSQ-8C, SATAQ4, GAD-7, and PHQ-9 among categories of TikTok usage.

**Figure 1.**
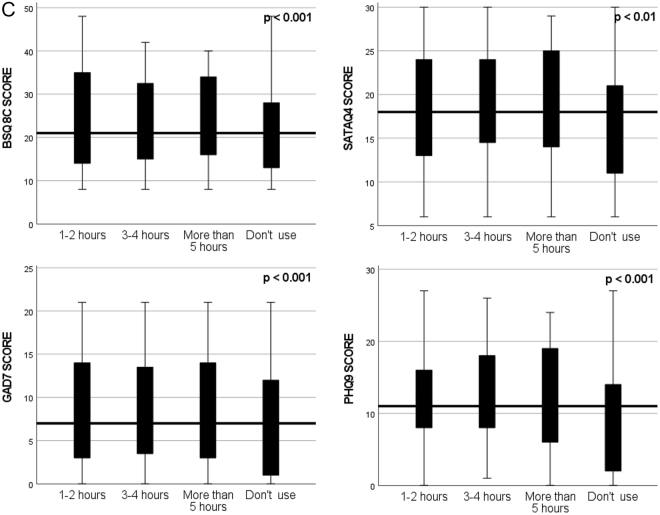
(C) Comparison of total score of BSQ-8C, SATAQ4, GAD-7, and PHQ-9 among categories of Snapchat usage.

Multinomial regression analysis was applied to find the effect of age, gender, content type, frequency of photo filter use, the photo filter (social media) platform, and the significance of photo filter on body dissatisfaction (BSQ-8C). The multinomial regression analysis identified gender and the perceived significance of photo filter use as significant predictors of body dissatisfaction. The negative B coefficient for gender (*B* = −3.778, *P* = 0.008) indicates that females had significantly higher BSQ-8C scores (indicating greater body dissatisfaction) compared to males. Similarly, attributing greater importance to photo filters was a significant predictor of higher body dissatisfaction (*B* = −6.383, *P* = 0.029).

## Discussion

Our findings corroborate a growing body of evidence linking photo-editing behaviors on social media with diminished self-perception and psychological distress. Specifically, increasing exposure to and the use of photo filters or photo-editing tools have been shown to reduce self-esteem and personal confidence, this is further mediated by increased self-objectification and frequent appearance comparisons on social media sites[13].

Fear of negative evaluation emerges as a central psychological driver behind filter use. A large-scale study (*N* = 2657) demonstrated that lower self-esteem predicted heightened fear of negative evaluation, which in turn increased the perceived importance of using Instagram filters, with fear of negative evaluation fully mediating this relationship[14].

Beyond individual psychological factors, broader patterns of social media engagement particularly when appearance focused are robustly associated with symptoms of anxiety and depression. It was found in a study of 763 adolescents and young adults, both general and appearance related social media utilization were significantly associated with depressive symptoms, social anxiety, and appearance as well as rejection sensitivity[15].

Self-objectification also plays a pivotal role in linking problematic social media use to body image disturbance and mental health outcomes. In a cross-sectional sample of 594 women, self-objectification mediated the relationship between social media usage and body image disturbances; importantly, physical activity attenuated this effect, while greater diet intensity exacerbated it. Furthermore, body image disturbance mediated associations between social media use and both depression and anxiety[16].

Complementing these findings, a systematic review of research through 2025 confirmed that exposure to visual, appearance-centric content on platforms such as Instagram and Snapchat is consistently tied to heightened self-objectification and body image concerns. The buffering possibilities of self-compassion interventions and media literacy interventions were also mentioned in the review[17].

These external research results are consistent with our data, which indicated that BSQ-8C (body dissatisfaction), SATAQ-4 (sociocultural appearance attitudes), GAD-7 (anxiety), and PHQ-9 (depression) had significant positive correlations, indicating that higher levels of body dissatisfaction are likely to be accompanied by internalized sociocultural ideals and increased anxiety and depression.

Moreover, our multinomial regression analysis identified gender and the significance attributed to photo filter use as the only significant predictors of body dissatisfaction and sociocultural appearance attitudes. This is in line with other wider evidence that women are more susceptible to internalizing appearance pressures and suffering related psychological distress.

A combination of these overlapping research streams, along with our own empirical evidence, supports the relevance of interventions designed to develop resilience and positive interactions with social media in young adults. The literature is supportive of strategies to improve media literacy and encourage self-compassion and integration of mental health support into digital services as potential ways to mitigate the negative effects on body image and mental health.

Beyond the direct associations observed, alternative explanations warrant consideration as well. One example would be individual personality traits such as high neuroticism, low self-esteem, or perfectionistic tendencies, to name a few, which render some users more susceptible to utilizing content focusing on appearance more frequently and idealizing the unrealistic beauty standards that it promotes. In this vein, those with existing or subclinical psychological vulnerabilities such as anxiety or depressive symptoms might be more prone to utilizing social media platforms to seek external validation and thus be exposed to greater amounts of appearance-based comparative information. Such individual psychological predispositions could thus help explain the noted correlations of social media use with body dissatisfaction and other mental health effects. Nevertheless, it is important to emphasize that the present study excluded participants with diagnosed psychological disorders to focus on identifying early, preclinical factors that may contribute to body image concerns and emotional distress before they progress to clinical levels. Additionally, we acknowledge that these potential confounding factors have not been directly assessed in this study and recommend that future longitudinal research explores their role in shaping the relationship between social media use, body image, and mental health.

This study has several limitations. Causal inference is not possible since the design is cross-sectional, and the long-term impact of the use of social media on the body image and mental health requires a longitudinal study in the future. Although participants with diagnosed mental health conditions were excluded, reliance on self-reported data may have introduced recall and social desirability biases. Lifestyle factors such as sleep, diet, and exercise were not controlled for and may have influenced the observed associations. Additionally, the study focused on platform usage frequency without accounting for engagement behaviors such as posting, commenting, or following influencers, which could provide deeper insight into the psychological impact of social media. Broader psychological traits, including personality factors as mentioned previously, were not assessed and should be considered in future research. The psychological outcomes observed in this study should be interpreted in light of inherent methodological constraints, including self-reporting and non-probability sampling. These factors, along with the cross-sectional design, underscore the need for longitudinal research to better understand the evolving impact of image-focused social media use on youth mental health.

## Limitations

This study relied on self-reported data, which may introduce recall or social-desirability bias. In addition, the use of convenience sampling may lead to selection bias, potentially limiting the generalizability of the findings. As this was a cross-sectional design, causality cannot be inferred, and the temporal direction of associations remains unclear. Future longitudinal and multi-site studies are recommended to validate these findings in broader populations.

## Conclusion

This study contributes to the accumulating literature on the connection between social media use – especially on photo-specific apps like TikTok, Snapchat, and Instagram – and increased body dissatisfaction and mental health problems, such as anxiety and depression. Findings indicate that females are more vulnerable to body dissatisfaction and the influence of sociocultural appearance pressures. To mitigate these adverse effects, we recommend the development of targeted interventions. This would involve for an educator and parent to put in place media literacy curricula that unstitch digital image manipulation and refute the standards of unrealistic beauty. For platform developers, ethical design changes, such as implementing disclaimers on filtered content or tools that will restrict the use of a platform by spending time on features that are appearance oriented. Finally, health care providers should consider routine screening for social media use patterns as part of mental health assessments for young people, and public health campaigns should promote help-seeking behaviors.

## Data Availability

Data available on request from the authors.

## Peer and provenance statement

Not commissioned, externally peer-reviewed.
